# Real-time sports injury monitoring system based on the deep learning algorithm

**DOI:** 10.1186/s12880-024-01304-6

**Published:** 2024-05-24

**Authors:** Luyao Ren, Yanyan Wang, Kaiyong Li

**Affiliations:** 1 Department of Physical Education, Nanjing Forestry University, Nanjing, Jiangsu 210037 China; 2 Department of Physical Education, Beijing Foreign Studies University, Beijing, 100089 China; 3 College of Physics and Electronic Information Engineering, Qinghai Nationalities University, Xining, Qinghai, 810007 China

**Keywords:** Sports injury monitoring, Deep learning algorithms, Machine learning, Medical applications

## Abstract

In response to the low real-time performance and accuracy of traditional sports injury monitoring, this article conducts research on a real-time injury monitoring system using the SVM model as an example. Video detection is performed to capture human movements, followed by human joint detection. Polynomial fitting analysis is used to extract joint motion patterns, and the average of training data is calculated as a reference point. The raw data is then normalized to adjust position and direction, and dimensionality reduction is achieved through singular value decomposition to enhance processing efficiency and model training speed. A support vector machine classifier is used to classify and identify the processed data. The experimental section monitors sports injuries and investigates the accuracy of the system’s monitoring. Compared to mainstream models such as Random Forest and Naive Bayes, the SVM utilized demonstrates good performance in accuracy, sensitivity, and specificity, reaching 94.2%, 92.5%, and 96.0% respectively.

## Introduction

Popular physiological responses to exercise injuries consist of muscle sprains, fatigue during exercise, cardiac overload, and tibial fibrosis. Effective identification and detection of the state of exercise of the body can protect against exercise injuries. It has been shown that optimizing training by identifying sports status can prevent, control, and monitor scattered sports injury risks. The use of effective training condition recognition and detection approaches can efficiently prevent fatality and shock from unexpected exercise injuries within a statistical range of 70–80%. By monitoring the risk of injury in sports, it is possible to find signs of injury and take effective action in a timely manner.

Currently, research in the field of sports injuries is extensive. Fonseca et al. focus on exploring methods for predicting sports injuries [1]. Bolling, Caroline, delves into the definition of sports injuries as perceived by athletes, coaches, and therapists [2]. Nielsen, Rasmus Oestergaard focus on treatment methods for different types of sports injuries and approaches to managing subsequent injuries in athletes [3]. Donaldson, Alex, examines the challenges faced in evidence-based sports injury prevention [4]. Park, So young systematically analyzes injury locations, rates, causes, and types among female athletes [5]. Despite some progress made in existing research, there are still many shortcomings in the monitoring and prevention of sports injuries, lacking comprehensiveness and precision.

The application of deep learning algorithms in the medical field has garnered widespread attention. Bai et al. propose machine learning-based methods for the identification of sports injuries [6]. Ba and Hongbing explore a deep learning system for sports injury medical rehabilitation based on image analysis [7]. Chang, Peter D, uses deep learning algorithms to detect complete tears of the anterior cruciate ligament [8]. Ozrazgat-Baslanti summarizes the research progress of deep learning in acute kidney injury related to the intensive care unit [9]. Rank, Nina introduces a deep learning-based algorithm capable of predicting acute kidney injury before the onset of symptoms and complications after surgery [10]. Despite the significant achievements of deep learning algorithms in the medical field, their application in sports injury monitoring remains relatively limited. This article addresses existing deficiencies in sports injury monitoring and proposes a new approach. Through steps such as video detection, human body keypoint detection, polynomial fitting analysis, mean calculation, normalization, singular value decomposition, and data dimensionality reduction, this paper establishes a comprehensive sports injury monitoring system. Compared to existing research, the proposed method is more comprehensive, accurate and practical, offering more effective solutions for the monitoring and prevention of sports injuries.

In this paper, the depth learning algorithm is used to study the real-time monitoring system of sports injuries. Firstly, the deep learning algorithm is applied to human target recognition, including motion detection and motion analysis. Then the research of sports injury real-time monitoring system is carried out, the design principle, the overall design, and the hardware design of the system are introduced, and the monitoring software is developed. In the experiment part, the real-time monitoring system of sports injury and the artificial experience analysis method are used to monitor the injury of running, aerobics and table tennis, and explore the monitoring accuracy of the monitoring system. The innovations in this article are as follows.

- Applying deep learning algorithms to real-time monitoring of sports injuries has improved detection accuracy.
- Deep learning-based monitoring systems have achieved high accuracy rates in activities such as running, aerobic exercise, and table tennis.
- The article presents a comprehensive design scheme for a sports injury monitoring system, integrating hardware and software, enabling the comprehensive monitoring and analysis of sports injuries.

## Human motion target motion recognition based on depth learning

**Fig. 1:**
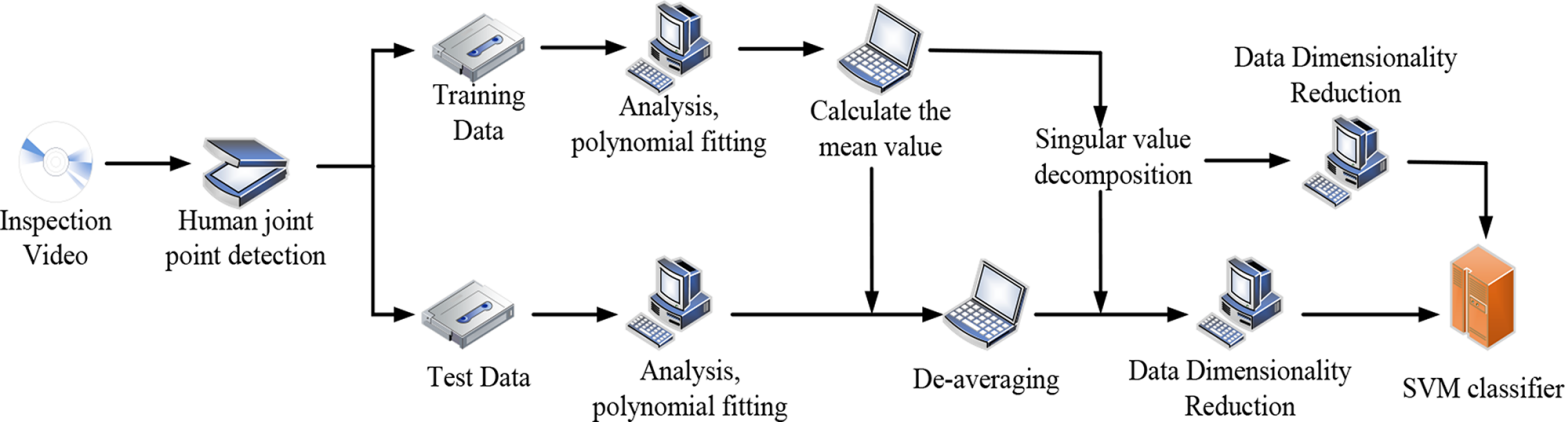
Human target movement recognition method

The research and application of machine learning in the medical field is becoming more and more extensive, and many achievements have been made, mainly in disease prediction, disease auxiliary diagnosis, disease prognosis evaluation, new drug development, health management, medical image recognition, etc. Deep learning is a new field of machine learning research. It can transform the original data from a simple nonlinear model to a higher level of abstract expression through the machine learning process, without human intervention, and then extract highly complex functional features by combining multilevel transformation learning. This is the main difference between deep learning and traditional machine learning. The commonly used deep learning algorithms in the medical field include convolutional neural networks, deep belief networks, deep neural networks, and recursive neural networks, which can be mainly used for disease diagnosis, drug development, medical image analysis, etc. In this paper, the depth learning algorithm is used to study real-time monitoring of sports injuries. First, human target action recognition is performed. The human target action recognition method is shown in Fig. 1.

The specific steps of this method are as follows.

1. Video detection: by examining video data, capture the movements of human targets
2. Human joint detection: use training and testing data to detect human joints.
3. Analysis, polynomial fitting: Perform polynomial fitting analysis on the detected joint data to extract motion patterns 4) between joints.
4. Calculate the mean: Calculate the mean of the training data as a reference point for data processing.
5. Demeaning: De-mean the original data to adjust data position and orientation. Use singular value decomposition to reduce the dimensionality of the data.
6. Data Dimension Reduction: Utilize dimensionality reduction techniques to improve data processing efficiency and model training speed.
7. SVM Classifier: Use the Support Vector Machine classifier to classify and identify processed data.

### Motion detection

The recognition of human actions is completed by determining the reliability map *S* of the body joints and the two-dimensional vector field *L* of the affinity domain of the joint points [11].

#### Collaborative detection and association

Collaborative detection and association involve identifying connectivity between human nodes and learning regions. First, the convolutional neural network is used to analyze the input graph, and the result of the association of features *F* is obtained as the input of each step. The first 10 layers of the VGG-19 network (Visual Geometry Group-19) are used to initialize and adjust the network. The network structure is divided into two iterative prediction branches. The first branch defines the two-dimensional characteristic graph *R* of body joints, and the second branch predicts the two-dimensional vector field *M* of the affinity of different body parts. The input of the first stage generates the confidence diagram $$ {R}^{1}$$ and vector field $$ {M}^{1}$$. In each subsequent prediction stage, the output of the previous stage is used as the input of this stage. The original characteristic diagram is *F* and the iterative calculation formula is as follows:

1 $$ {R}^{t}={\rho }^{t}({F}^{t},{R}^{t-1},{M}^{t-1})$$

2 $$ {M}^{t}={\rho }^{t}({F}^{t},{R}^{t-1},{M}^{t-1})$$

Among them, ${\rho }^{t}(\bullet )$ represents the iterative calculation of branches at each stage of the neural network. Formula 1 iteratively computes confidence maps and vector fields using convolutional neural networks to accurately recognize human actions. Formula 2 evaluates the accuracy of predicted confidence maps to guide model optimization.

A loss function $$ {L}_{2}$$ is added at the end of each step to provide more accurate prediction results. In order to deal with the problem that some data in the dataset cannot be completely marked, the weight of each loss function is used to form the following loss function:

3 $$f_R^t = \mathop \sum \limits_{j = 1}^J \mathop \sum \limits_q {\rm{w}}\left( q \right).\parallel S_j^tq - S_j^*\left( q \right)\parallel _2^2$$

4 $$f_{\rm{M}}^{\rm{t}} = \mathop \sum \limits_{c = 1}^C \mathop \sum \limits_q {\rm{w}}\left( q \right).\parallel S_c^tq - S_c^*\left( q \right)\parallel _2^2$$

Among them, ${S}_{j}^{*}\left(q\right)$ and ${S}_{c}^{*}\left(q\right)$ are the real marked values, and w (q) is 0 and 1, and when the point is marked it is 1. If the point is marked, it is 1, otherwise it is 0, so as to avoid punishing the model when the sample is positive. Formula 3 calculates local affinity field associations to ensure connectivity among all joints, enhancing model robustness and accuracy. Formula 4 maps the node detection results to the confidence maps for further joint detection and action recognition. The final objective function is in the following form:

5 $$ \text{f}={\sum }_{\text{t}=1}^{\text{T}}({\text{f}}_{\text{R}}^{\text{t}}+{\text{f}}_{\text{M}}^{\text{t}})$$

Formula 5 optimizes confidence maps to improve node detection accuracy and robustness.

#### Joint point confidence map detection

When nodes are detected in the input image, the convolution neural network operation can detect the node map, which is called the confidence map. This work uses several training steps. Each step uses the results of the previous training step and optimizes them to bring the final results closer to the target value [12].

First, the confidence map of the nth individual is $$ {s}_{j,n}^{*}\left(q\right)$$, and $$ {x}_{j,n}$$ is the true value of the nth individual at the joint point j. Then the predicted value of the point *q* can be expressed as a Gaussian function in the following form:

6 $$s_{j,n}^*\left( q \right) = exp - {{\parallel q - {x_{j,n}}\parallel _2^2} \over {{\sigma ^2}}}$$

Formula 6 predicts node positions using Gaussian functions to improve node detection precision. For each different real point *x*, there is a symmetrical curve at point *q*, and the maximum operation method is as follows:

7 $$ {s}_{j}^{*}\left(q\right)={}_{n}{}^{max}{s}_{jn}^{*}\left(q\right)$$

Formula 7 selects the maximum confidence value as the node detection result, improving the stability of the result.

#### Association of the local affinity domain

Once human joints are detected, all joints must be connected. If the connection only considers the validity of two detection points, when there is more than one point, an incorrect connection would occur.

$$ {x}_{{j}_{1},n}$$ and $$ {x}_{{j}_{2},n}$$ represent the actual positions of joint point $$ {j}_{1}$$ and joint point $$ {j}_{2}$$ of the nth person on limb *c*, so the form of the vector field of the point *q* is:

8 $$ {L}_{c,n}^{*}\left(q\right)=\left\{\begin{array}{c}V, q on c\\ o, other\end{array}\right.$$

Among them, ${\rm{V}} = \left( {{x_{{j_1},n}} - {x_{{j_2},n}}} \right)/\parallel {x_{{j_1},n}} - {x_{{j_2},n}}{\parallel _2}$ represents the unit vector of the direction of the limb. Formula 8 computes vector fields between nodes based on the direction of the limb, connecting joints of different parts of the body. Finally, the affinity fields of all individuals at point *q* are averaged as follows:

9 $$ {L}_{c}^{*}\left(q\right)=\frac{1}{{n}_{c}\left(q\right)}\sum _{n}{L}_{c,n}^{*}\left(q\right)$$

Formula 9 calculates the reliability of the connections between joints through the affinity field, ensuring the accuracy and rationality of the connection. $$ {n}_{c}\left(q\right)$$ represents the number of all nonzero vectors of *the point q.* For any two joint points $$ {d}_{{j}_{1}}$$ and $$ {d}_{{j}_{2}}$$, the reliability *E* of the affinity field relationship is calculated by the distance between the two points:

10 $${\rm{E}} = \mathop \smallint \limits_{{\rm{u}} = 0}^{{\rm{u}} = 1} {{\rm{L}}_{\rm{c}}}\left( {{\rm{q}}\left( {\rm{u}} \right)} \right).{{{d_{{j_2}}} - {d_{{j_1}}}} \over {\parallel {d_{{j_2}}} - {d_{{j_1}}}_2\parallel }}{\rm{du}}$$

11 $$ \text{q}\left(\text{u}\right)=(1-\text{u}){\text{d}}_{{\text{j}}_{1}}+\text{u}{\text{d}}_{{\text{j}}_{2}}$$

Formula 10 averages the affinity fields of all individuals to consider the connection accuracy in a comprehensive way. Formula 11 evaluates the reliability of the connection relationships between joints through the calculation of the reliability of the affinity field. In the calculation, the integral is approximately solved by the sum of the uniformly distributed values of the discretized *u*.

### 2.2 Motion analysis

Because the position of the human body changes constantly during the sampling process, it is necessary to standardize the test data. As the width/height ratio of the human shoulder is 1:4, the *x* and *y* values of each joint point are normalized to the range of [-200,200] and [-800,800], that is:

12 $$ {x}_{i}=400\times \frac{{x}_{oi}-{x}_{min}}{{x}_{max}-{x}_{min}}-200$$

13 $$ {y}_{i}=1600\times \frac{{y}_{oi}-{y}_{min}}{{y}_{max}-{y}_{min}}-800$$

Formula 12 standardizes the sampled data to meet the requirements for subsequent motion analysis. Formula 13 utilizes the orthogonal Procrustes problem to optimize the spatial coordinate fitting effect of the model. Among them, ($$ {x}_{oi}$$,$$ {y}_{oi}$$) and ($$ {x}_{i}$$,$$ {y}_{i}$$) respectively represent the point coordinates before and after normalization, and $$ {x}_{max}$$, $$ {x}_{min}$$, $$ {y}_{max}$$ and $$ {y}_{min}$$ represent the maximum and minimum values of the sample point coordinates.

#### Proctor analysis

Mathematically, the orthogonal Proctor problem is to solve an orthogonal matrix $$ {R}_{0}$$ so that $$ {AR}_{0}$$ is as close to *B* as possible. *A* and *B* are the space coordinates of two objects, that is, to solve the convergence problem.

14 $${f_0}\left( R \right) = {}_{\rm{R}}^{min}\parallel {\rm{A}}{{\rm{R}}_0} - {\rm{B}}{\parallel _{\rm{F}}}$$

Among them, $\parallel .{\parallel _F}$ is the *F* norm. Formula 14 determines the centroid of samples for reference in subsequent translation operations.

A. Translation.

First, the mass center of the sample is determined, which can be expressed by the following formula:

15 $$ (\stackrel{-}{\text{x}},\stackrel{-}{\text{y}})=(\frac{1}{\text{N}}\sum _{\text{i}=1}^{\text{N}}{\text{x}}_{\text{i}},\frac{1}{\text{N}}\sum _{\text{i}=1}^{\text{N}}{\text{y}}_{\text{i}})$$

Formula 15 performs translation operations on original coordinate points to correct data position and orientation. Among them, *N* represents the number of corresponding points in the sample, so all data points must undergo a translation operation:

16 $$ ({\text{x}}_{\text{i}}^{{\prime }},{\text{y}}_{\text{i}}^{{\prime }})=({\text{x}}_{\text{i}}-\stackrel{-}{\text{x}},{\text{y}}_{\text{i}}-\stackrel{-}{\text{y}})$$

Formula 16 computes the scaling ratios in the X and Y directions to ensure uniform data proportions. Among them, $({\text{x}}_{\text{i}}^{{\prime }},{\text{y}}_{\text{i}}^{{\prime }})$ represents the position of the point after translation.

B. Scaling.

The scaling ratio in the *X* direction and the scaling ratio in the *Y* direction must be determined, respectively, that is, *F* norm $$ {S}_{Fx}$$ and $$ {S}_{Fy}$$:

17 $$ {S}_{Fx}=\sqrt{\frac{1}{\text{N}}\sum _{\text{i}=1}^{\text{N}}{\widehat{{\text{x}}_{\text{i}}}}^{2}}$$

18 $$ {S}_{Fy}=\sqrt{\frac{1}{\text{N}}\sum _{\text{i}=1}^{\text{N}}{\widehat{{\text{y}}_{\text{i}}}}^{2}}$$

Formula 17 uses the least squares method to find the optimal rotation angle based on the distance between the data points, optimizing data fitting. Formula 18 reduces data errors and improves motion estimation accuracy through calibrated data from Procrustes analysis.

C. Rotation:

The Proctor distance is defined as:

19 $$ {P}_{d}^{2}=\sum _{i=1}^{N}[{({x}_{il}-{x}_{i0})}^{2}+{({y}_{il}-{y}_{i0})}^{2}]$$

Formula 19 fits human joint data using a polynomial fitting program as a basis for subsequent motion estimation. Among them, $$ ({x}_{i0},{y}_{i0})$$ and $$ ({x}_{il},{y}_{il})$$ are model points and sample points, respectively. The best rotation can be found with the least square method θ. The Proctor distance $$ {P}_{d}^{2}$$ between the sample and the model is minimized.

Calibration of the data after Proctor analysis can reduce the error caused by camera position and the different distance between the detected human body and camera, making the final estimate result more reasonable [13].

#### 2.2.2 Polynomial fitting

**Fig. 2:**
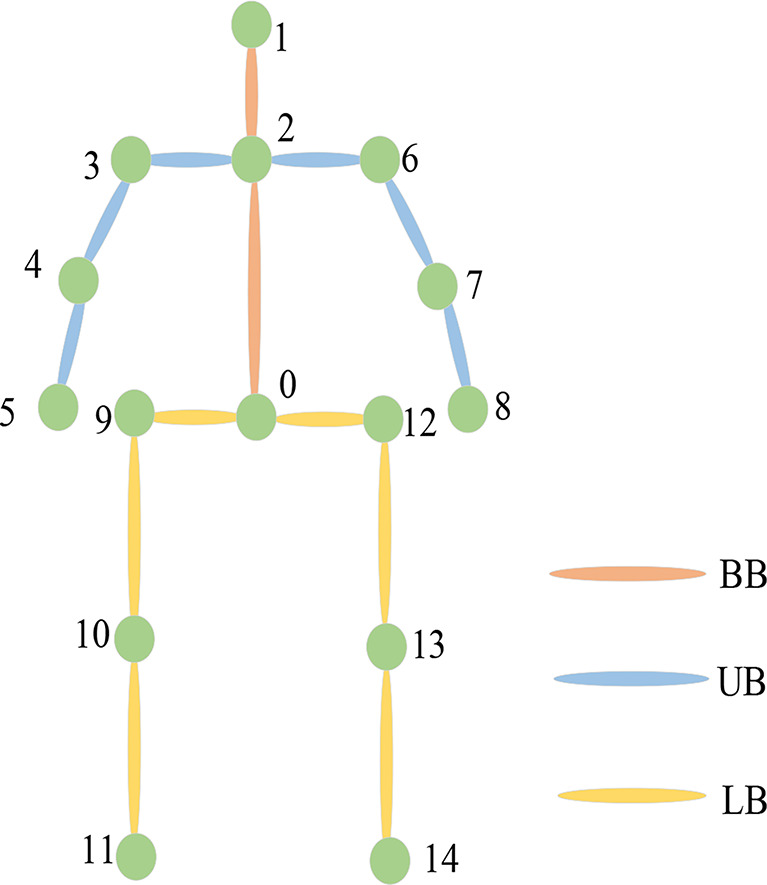
Human Joint Point Model

A human body model can generally be represented as a simple rigid body connected by a series of joint points, as shown in Fig. 2.

14 common points were selected as data for further analysis. If the human body is considered to be a layered arrangement of joints, it can be divided into three parts. BB connects three joints --0, 1 and 2; UB connects seven points --5, 4, 3, 2, 6, 7 and 8; LB connects seven points --11, 10, 9, 0, 12, 13, and 14. In this way, overlapping joints can be analyzed by parts. This paper uses the polynomial fitting program of human joint data as a basis for subsequent motion estimation.

Polynomial fitting plays an important role in numerical analysis. It gives a smooth function that is easy to handle and calculate. In addition, polynomial fitting can also provide accurate convergence when approximating numerical data, but polynomial fitting can only provide accurate approximation within a short interval. The general formula for polynomial fitting is given by the formula:

20 $${\rm{p}}\left( {\rm{x}} \right) = {{\rm{c}}_0} + {{\rm{c}}_1}{\rm{x}} + ... + {{\rm{c}}_{\rm{n}}}{{\rm{x}}^{\rm{n}}}$$

Formula 20 provides a smooth function to approximate numerical data and ensures accurate fitting. Among them, ${\text{c}}_{\text{n}}$ represents the fitting coefficient of the polynomial.

The polynomial fitting in this paper assumes that all coordinate points are located in the x-y plane, so the origin is located at the midpoint of the two femurs. As described in the article, the body joint points are divided into three parts for analysis, namely BB, UB and LB, and each part is polynomial fitted from the X axis direction and Y axis direction, respectively.

This paper introduces the concept of goodness of fit $$ {R}_{R}^{2}$$ of curves:

21 $$ {R}_{R}^{2}=1-\frac{{\sum }_{i=1}^{n}{({y}_{Ni}-\widehat{{y}_{Ni}})}^{2}}{{\sum }_{i=1}^{n}{({y}_{Ni}-\stackrel{-}{{y}_{Ni}})}^{2}}$$

22 $$ \stackrel{-}{{y}_{Ni}}=\frac{1}{{n}_{0}}\sum _{i=1}^{{n}_{0}}{y}_{Ni}$$

Formula 21 evaluates the effect of data fitting through the goodness of fit of curve fitting. Formula 22 performs dimensionality reduction on data through principal component analysis (PCA) to reduce data dimensions. Among them, $$ {y}_{Ni}$$ represents the value of the original node on the *x* and *y* axes, $$ \widehat{{y}_{Ni}}$$ represents the value after polynomial fitting, $$ \stackrel{-}{{y}_{Ni}}$$ represents the sample mean value, and $$ {n}_{0}$$ represents the number of fitting data.

A good fitting value $$ {R}_{R}^{2}$$ is in the range of [0,1]. If the value is closer to 1, it indicates that the fitting effect of the data is better and vice versa.

#### Reduced dimension data and action analysis

This paper analyzes the post polynomial fitting data of human joints on the x-axis and y-axis, respectively, and then conducts Principal Component Analysis (PCA) on these data to reduce the dimensions of the data. This method performs an orthogonal decomposition of the features of the sample data to obtain the main components of the converted data information [14].

The main steps of dimension reduction through PCA can be divided into five stages.

A. The average value of all training samples is calculated:

23 $$ \stackrel{-}{X}=\frac{1}{{n}_{i}}\sum _{i=1}^{{n}_{i}}{X}_{i}$$

B. The covariance matrix of the sample is calculated:

24 $$ \text{A}={\sum }_{\text{i}=1}^{{\text{n}}_{\text{i}}}{({\text{X}}_{\text{i}}-\stackrel{-}{\text{X}})}^{\text{T}}({\text{X}}_{\text{i}}-\stackrel{-}{\text{X}})$$

C. The eigenvalue of the sample covariance matrix is decomposed, and the K-L transformation matrix is found:

25 $$ \text{A}\times \text{V}=\text{V}\times \text{D}$$

D. According to the required precision, the PCA dimension reduction matrix is constructed by selecting the principal component of the K-L change:

26 $$ {M}_{T}=\sum _{i=1}^{{n}_{i}}({\text{X}}_{\text{i}}-\stackrel{-}{\text{X}})\times {\text{V}}_{\text{P}\text{C}\text{A}}\times {\text{D}}_{\text{P}\text{C}\text{A}}$$

E. The data are dimensionally reduced:

27 $$ \text{M}={({\text{X}}_{\text{i}}-\stackrel{-}{\text{X}})}^{\text{T}}\times {\text{M}}_{\text{T}}$$

Among them, $$ {X}_{i}$$ represents available data and is a vector; $$ {V}_{PCA}$$ and $$ {D}_{PCA}$$ are eigenvalues and eigenvector matrices after selecting the corresponding principal components; $$ {M}_{T}$$ is the reduced dimension matrix and *M* is the reduced dimension data.

Formula 23 computes the average of all training samples as a benchmark for reducing the dimension of the data. Formula 24 calculates the covariance matrix of samples for feature decomposition in PCA dimensionality reduction. Formula 25 decomposes the eigenvalues of the covariance matrix to obtain the principal component transformation matrix. Formula 26 constructs a PCA dimensionality reduction matrix by selecting the principal components according to the required precision, reducing the dimensions of the data. Formula 27 completes the reduction of the dimensionality of the data to obtain the processed data for subsequent motion analysis. The training and test data processing methods are slightly different. The training data are averaged, that is, the sample average, and all training data are subtracted from the sample average. Then the covariance matrix is calculated to determine the eigenvalues and eigenvectors, and the reduced-dimension matrix is obtained through the contribution calculated eigenvectors. The final training data is obtained by multiplying the de-averaged training data and the reduced dimension matrix [15].

In damage monitorThis article introduces a model for ing systems that use the Support Vector Machine (SVM) technique. The input layer, decision function, kernel function, and support vectors make up the model. Pre-processed data, including mobility statuses and physiological markers, are received at the input layer. The decision function categorizes the input data and makes predictions based on the kernel function output. Support vectors, pivotal data points selected during training, determine classifier boundaries and performance. Through iterative optimization, the model accurately identifies and predicts motion injuries.

This article uses the SVM model for action recognition tasks. The structure of the SVM model consists of an input layer, a hidden layer, and an output layer. The input layer receives data that has been polynomially fitted, the hidden layer performs feature extraction using the ReLU activation function, and the output layer employs the softmax function to output the probability distribution of various action categories. During the training process, the stochastic gradient descent (SGD) optimizer is utilized with a learning rate set to 0.001. Training and validation sets are divided using random sampling, with 70% of the data used for training and 30% for validation.

## Evaluation of sports injury real-time monitoring system

### Design principle of sports injury real-time monitoring system

A real-time sports injury monitoring system consists of two parts: a portable mobile terminal and a main monitoring system.

The portable mobile software device is used to describe the state of the human body in the process of movement and the human body in the state of motion. The system is responsible for acquiring real-time data of human heart rate, pulse, cardio pulmonary function, heart rate, muscle tension, pulmonary function, and other parameters during exercise as baseline analysis data to determine status and injury risk. In the long-term test, physiological indicators of individual training performance are completely recorded, and the data can be easily analyzed and processed for the sports injury risk assessment experiment. The data processing of experimental results is combined with an expert system to describe physiological performance and state monitoring. If abnormal data is detected, the simulator will notify by alarm [16].

**Fig. 3:**
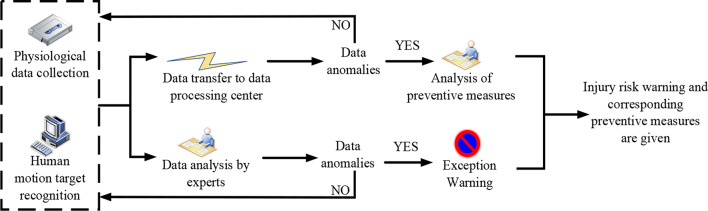
The principal implementation process of an injury monitoring system based on human motion state recognition

The main control monitoring system can identify the target action of human movement and analyze the state of human movement and injury risk data in real time. If the patient is found to be really dangerous, the information would be immediately transmitted through the expert system and appropriate warnings and preventive measures would be issued. According to the above design analysis, a damage monitoring system based on human motion state recognition. is established. The principle of process realization is shown in Fig. 3.

The specific process illustrated in Fig. 3 is as follows: First, the system collects physiological data, including parameters such as heart rate, pulse, and cardiorespiratory function of the human body. Next, the system identifies the target movements of the human body through the main control monitoring system and analyzes the movement status and risk of injury data in real-time. Once a patient is found to be at risk, the system immediately communicates this information through an expert system. The expert system analyzes the data, identifies abnormal conditions, and proposes the corresponding preventive measures. In the event of abnormal conditions, the system sends an alert, providing warnings about injury risks and the corresponding preventive measures to reduce or avoid the occurrence of injuries.

### Overall design of the system

Based on the above analysis of the design principles of the athlete injury risk monitoring system, the overall design of the system is described. The main functions of the system include detecting the physiological state of people, identifying their movement, and processing information. Hardware includes data acquisition module, human motion recognition module, information processing module of mobile terminal software, main control module, analog to digital converter (ADC) module, and injury risk analysis module.

**Fig. 4:**
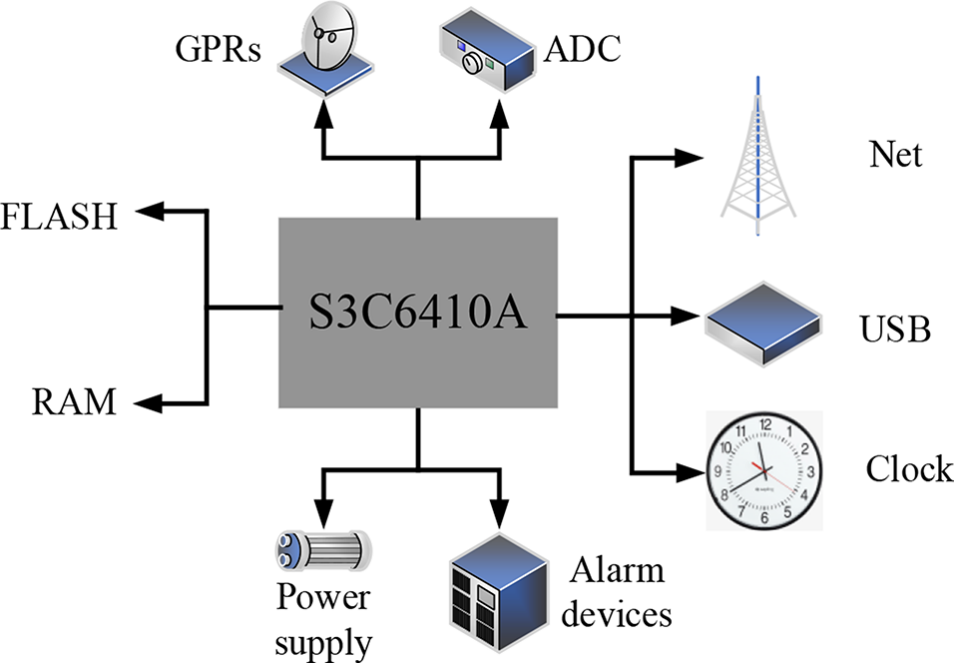
The overall design structure of the sports injury risk monitoring system

The hardware and peripheral circuit designs for the motion injury risk monitoring system are shown in Fig. 4, which also depicts the system’s overall architecture. The data collecting module, human motion recognition module, information processing module for mobile terminal software, primary control module, analog-to-digital converter (ADC) module, and injury risk analysis module are all included in the hardware part. Peripheral circuit designs encompass the Universal Serial Bus (USB) module, clock circuit, network communication module, general wireless data packet communication module, ADC circuit, and sensor adjustment circuit. Key functionalities include detecting human physiological status, identifying human motion, and processing information. The entire system achieves monitoring and analysis of motion injury risks through collaborative operation of these modules, thereby providing timely alerts and preventive measures.

### Hardware design of the system

S3C6410 is the main processor of the human motion data acquisition and analysis system. The hardware design includes the following parts: USB module design, timing circuit design, network communication module design, general wireless packet industrial communication module design, ADC circuit design, and sensor regulation circuit design.

#### Circuit of the USB module

The design of this module is to use the terminal interface function of the mobile software to achieve interface compatibility and data transmission of the sports injury risk monitoring system. The system designs a circuit from USB module to sensor output, and uses internal circuits (such as filter, converter, amplifier, etc.) to provide original data for sports injury monitoring. The USB interface design uses parallel peripheral interfaces to form the clock interrupt interface of the human sports injury risk monitoring system. The power consumption of the sports injury risk monitoring system is very low [17].

#### Clock circuit

The clock circuit of the human sports injury risk monitoring system is used to interrupt the clock frequency of the physiological state information of the human sports injury risk monitoring system. According to the design requirements of the Android system for mobile handheld devices, the sports injury information logic controller automatically adjusts the energy spectrum detection gain according to signal size and selects the BOOTM pin [0: 3] to set the charging mode [18].

#### The network communication module

Net network communication module is the core of the entire system. First, the network communication module of the human sports injury risk monitoring system was developed on the Android mobile terminal, and the portability of the operating system and the compatibility of the device ports were realized through the WiFi interface. The online sports injury risk identification and evaluation records of the expert system were used.

#### ADC circuit design

The ADC circuit is designed for the storage, allocation, and function sampling of human sports injury risk monitoring information, and uses MC7660 as the ADC chip.

#### Conditioning circuit design

The sensor conditioning circuit of the human motion injury monitoring system can realize the signal conditioning function. When recognizing human motion status, a signal conditioning circuit must be developed for filtering and amplification to improve recognition ability. The signal conditioning circuit uses S3C6410 as the main processor of the system. Since the human body beats 60 to 100 times per minute during movement, the signal conditioning circuit is designed to amplify the signal by more than 100 times. The signal filter is a low-pass filter with a cut-off frequency higher than 20 Hz [19].

### Software development of the system

In this paper, software is developed for the hardware part of the injury risk monitoring system based on human motion detection. When developing the software for the portable injury risk monitoring system, ARM1176JZF-S is used as the main processing core and the WiFi interface is used to achieve portability of the operating system and compatibility of device transplantation. First, a working directory is created and then a cross-compilation check of the WiFi interface is carried out. Finally, risk monitoring and evaluation is carried out in the new compiler [20].

## Real-time monitoring experiment of sports injury

To assess the effectiveness of real-time sports injury monitoring, we selected 30 subjects for testing. They wore our developed portable sports injury risk monitoring system while running, aerobic exercise, and table tennis, and collected motion data over the course of a week. Ten subjects participated in each type of activity. We compared the results of the evaluation of sports injuries between the system described in this article and traditional methods of manual expert analysis, using the precision of the risk monitoring signals as the test metric.

### Running

**Fig. 5:**
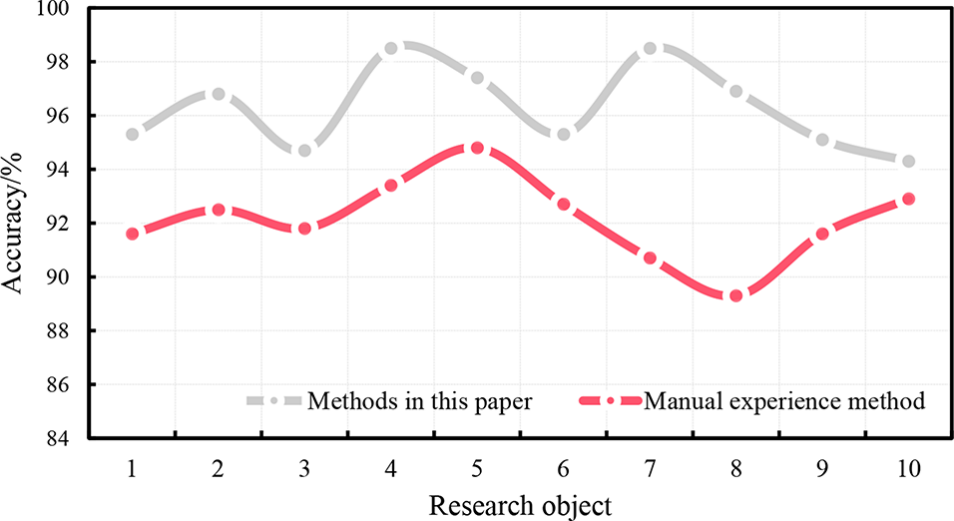
Accuracy of Running Injury Risk Monitoring

Ten subjects were monitored for running for one week. The precision of the sports injury risk monitoring of this system and the traditional artificial experience analysis method are shown in Fig. 5.

It can be seen from the data that the accuracy of the real-time sports injury monitoring system in this paper for the risk monitoring of running injury of 10 research objects is more than 94%, and the accuracy of the risk monitoring of running injury based on the traditional manual experience analysis method is between 89% and 95%. For sports injuries in running, the monitoring accuracy of the monitoring system in this paper is higher than that of traditional empirical analysis, which shows that the application of the deep learning algorithm in machine learning to the monitoring of running sports injuries can better monitor human condition during running, and timely carry out damage warning and prevention.

### Aerobics

**Fig. 6:**
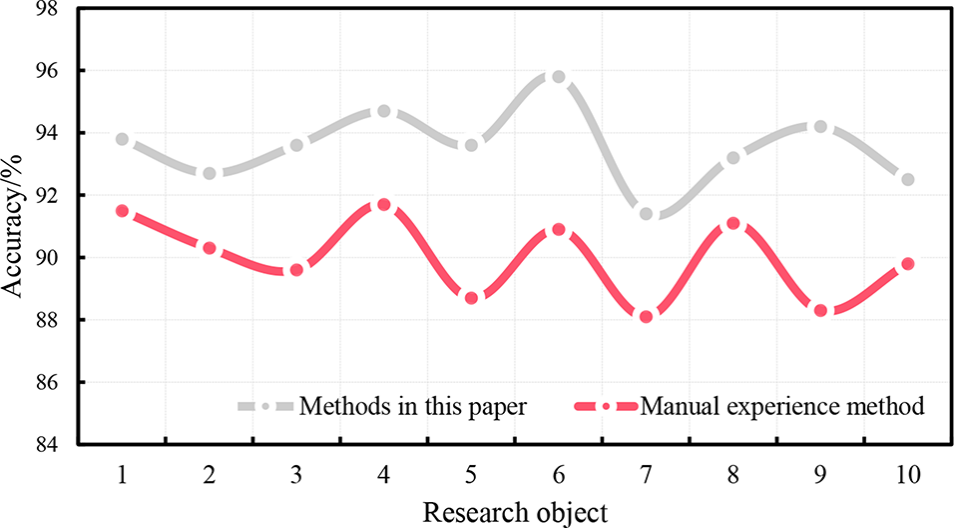
Accuracy of Monitoring Sports Injury Risk in Aerobics

Ten subjects were monitored for aerobics for one week, and the precision of the sports injury risk monitoring of this system and the traditional artificial experience analysis method is shown in Fig. 6.

It can be seen from the data that when 10 research objects are engaged in aerobics, the accuracy of the real-time monitoring system of sports injury in this article is 91-96%, and the accuracy of the risk monitoring of sports injury in aerobics based on the traditional manual experience analysis method is 88-92%. By comparing the data, it is found that the monitoring accuracy of the monitoring system in this paper is higher than that of the traditional empirical analysis, which shows that the application of the deep learning algorithm in machine learning to the monitoring of aerobic sports injury can better monitor the human state during aerobic sports, and timely carry out injury warning and prevention.

4.3 Table tennis.

**Fig. 7:**
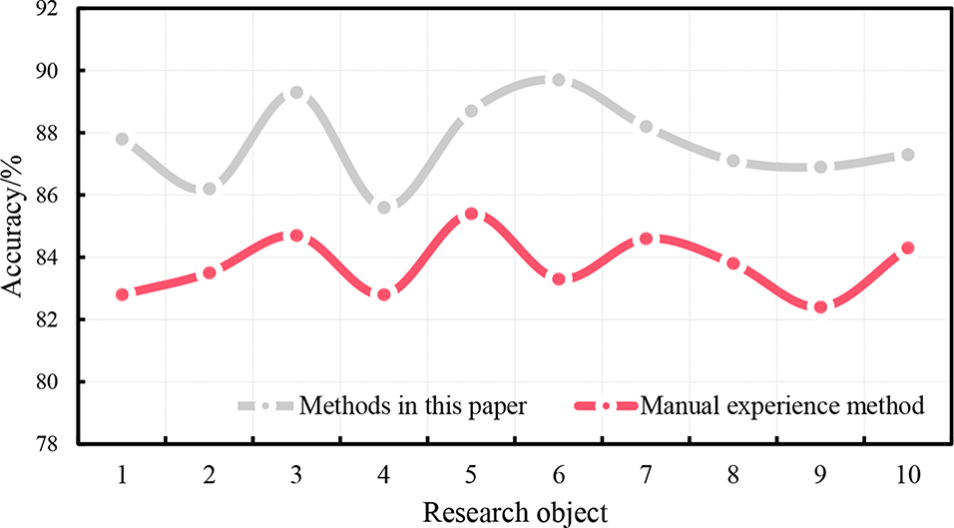
Monitoring the precision of the risk of injury in table tennis

Ten subjects were monitored for table tennis for one week. The accuracy of sports injury risk monitoring of this system and the traditional artificial experience analysis method are shown in Fig. 7.

It can be seen from the data that the precision of the sports injury risk monitoring of the real-time sports injury monitoring system in this article is 85-90%, and the precision of the table tennis injury risk monitoring based on the traditional artificial experience analysis method is 82-86%. For sports injuries in table tennis, the monitoring accuracy of the monitoring system in this paper is higher than that of the traditional empirical analysis, which shows that the application of the depth learning algorithm in machine learning to the monitoring of sports injuries in table tennis can better monitor the state of the human body during sports, and timely carry out injury warning and prevention.

### Comprehensive analysis

The monitoring results of the three sports modes are comprehensively analyzed and the results are shown in Table 1.

**Table 1:** Comprehensive analysis of results of sports injury monitoring

Exercise style	Deep learning monitoring system	Human experience analysis
Running	96.28	92.13
Aerobics	93.55	90
Table tennis	87.68	83.76
Comprehensive results	92.5	88.63

The depth learning algorithm sports injury monitoring system has an average accuracy rate of 96.28% for real-time monitoring of running injuries, 93.55% for real-time monitoring of aerobics injuries, and 87.68% for real-time monitoring of table tennis injuries. The average accuracy of the artificial experience analysis method for real-time monitoring of running injury is 92.13%, the average accuracy for real-time monitoring of aerobic injury is 90%, and the average accuracy for real-time monitoring of table tennis injury is 83.76%. From comprehensive data, it can be calculated that the average accuracy of the depth learning algorithm sports injury monitoring system is 92.5%, and the average accuracy of the artificial experience analysis method is 88.63%. Applying the depth learning algorithm in machine learning to sports injury monitoring has higher accuracy. Compared to the traditional artificial experience analysis method, the accuracy increases by 3.87%, which can better monitor sports injuries and prevent injuries. This shows that machine learning algorithm has high performance computing power and has broad applications prospects in the medical field.

The model in this paper is compared with other related algorithms in the analysis of sports injuries, and the results are shown in Table 2. In this study, all models were evaluated using the same dataset. The data set comprises diverse sports injury cases from various sports events and training scenarios. It contains comprehensive physiological parameters as well as motion data gathered during athletic events. SVM models were trained and assessed based on a data set of 1000 samples, each of containing movement data collected by wearable sensors together with physiological features including heart rate, blood pressure, and muscle tension. Similar assessments of other models covering different features including sports injuries, physiological factors, and motion characteristics were conducted on the same dataset. The efficiency of the suggested models in the study of sports injuries was confirmed by a thorough review of this dataset, which included an analysis of each algorithm’s performance.

**Table 2:** Model comparison result

Algorithm	Precision (%)	Sensitivity (%)	Specificity (%)
SVM	94.2	92.5	96.0
Random Forest	91.5	88.8	93.2
Naive Bayes	87.3	84.6	90.1
Support Vector Regression	92.8	90.2	94.3
AdaBoost	89.6	86.9	91.8
Logistic Regression	88.9	86.3	90.7
K-Nearest Neighbors	88.5	85.0	91.2
Decision Tree	90.1	88.0	92.3
Gaussian Process Regression	93.4	91.7	94.9
Support Vector Machine Regression	92.3	89.6	93.7

With results of 94.2%, 92.5%, and 96.0%, accordingly, Table 2 shows the way the SVM algorithm operates with respect to of accuracy, sensitivity, and specificity. Among the other models, the Gaussian process regression exhibits the highest precision, reaching 93.4%. However, the SVM model remains competitive in terms of precision and specificity while also offering faster training speeds and better interpretability. Therefore, for sports injury analysis, the SVM model remains a reliable choice. Compared to existing methods that use similar data, such as MRI [21], the system proposed in this document offers greater real-time capability and specificity, allowing faster and more accurate injury analysis and risk warning, thus protecting the health of athletes.

## Conclusions

Based on the high computing performance of machine learning technology, this paper used the deep learning algorithm in machine learning to study the real-time sports injury monitoring system. In this paper, the motion depth learning algorithm was used to recognize actions of human motion targets. Then a real-time monitoring system for sports injury was established, including human physiological data monitoring and human motion target action recognition. In the experiment part, the monitoring effect of the monitoring system was studied. The experimental results showed that the motion injury monitoring system based on the depth learning algorithm has higher monitoring accuracy than the traditional artificial experience analysis method, which shows that the machine learning algorithm can be better applied to the medical field.

## Data Availability

Data sets were not generated or analysed during the current study.
